# Neoadjuvant imatinib treatment and laparoscopic anus-preserving surgery for a large gastrointestinal stromal tumor of the rectum

**DOI:** 10.1186/s12957-016-0837-1

**Published:** 2016-03-08

**Authors:** Kennoki Kyo, Masaki Azuma, Kazuya Okamoto, Motohiro Nishiyama, Takahiro Shimamura, Atsushi Maema, Hitoshi Kanamaru, Motoaki Shirakawa, Toshio Nakamura, Kazuya Shinmura, Kenji Koda, Hidetaro Yokoyama

**Affiliations:** Department of Surgery, Fujieda Municipal General Hospital, 4-1-11 Surugadai, Fujieda, Shizuoka 426-8677 Japan; Department of Pathology, Hamamatsu University School of Medicine, 1-20-1, Handayama, Higashi-ku, Hamamatsu, Shizuoka 431-3192 Japan; Department of Pathology, Fujieda Municipal General Hospital, 4-1-11 Surugadai, Fujieda, Shizuoka 426-8677 Japan

**Keywords:** Gastrointestinal stromal tumor, Neoadjuvant treatment, Rectum, Imatinib, Laparoscopic surgery

## Abstract

**Background:**

Resection of a gastrointestinal stromal tumor (GIST) of the rectum can be difficult because of the particular location in the pelvis, and a large rectal GIST often requires abdominoperineal resection. Recent reports demonstrate that neoadjuvant imatinib treatment improves surgical outcomes in patients with a rectal GIST, and there are only a few reports of the effectiveness of laparoscopic surgery for a rectal GIST.

**Case presentation:**

A 46-year-old man was found to have a rectal GIST that measured 80 mm and was located on the anterior wall of the lower rectum. After 6 months treatment with imatinib, the tumor decreased in size to 37 mm, and laparoscopic low anterior resection was performed. The patient is currently alive without any evidence of recurrence 37 months after surgery.

**Conclusions:**

Neoadjuvant imatinib should be a treatment of choice for a large rectal GIST. When marked tumor shrinkage is achieved, laparoscopic surgery may be the preferred procedure.

## Background

Gastrointestinal stromal tumors (GISTs) are rare tumors with an estimated incidence of 1.5/100,000/year but are the most common mesenchymal tumors of the gastrointestinal tract [1]. They are most commonly found in the stomach (60 %) and small intestine (35 %), and <5 % arise in the rectum [2]. Although complete surgical excision with histologically negative margins is the primary treatment of choice for localized GISTs, resection of rectal GISTs can be difficult in the narrow pelvic space, and a large rectal GIST has a risk of rupture during surgery and often requires abdominoperineal resection. GISTs express KIT proto-oncogenic receptor tyrosine kinase and commonly harbor activating mutations in the KIT gene [3]. Imatinib is an inhibitor of tyrosine kinases including KIT, and targets the aberrant signaling pathways that are critical for tumor cell proliferation and survival, thus showing anti-tumor activity [4]. The effectiveness of imatinib in the treatment of GISTs was first described in 2001 [5], and the remarkable response to imatinib in unresectable or metastatic GISTs has led to the neoadjuvant treatment strategy for locally advanced GISTs [6, 7]. Despite the excellent view of laparoscopy, there are only a few that reported cases of its use in surgery for rectal GIST [8, 9]. We report here a patient with a large rectal GIST who safely underwent laparoscopic anus-preserving surgery after neoadjuvant imatinib treatment.

## Case presentation

**Fig. 1:**
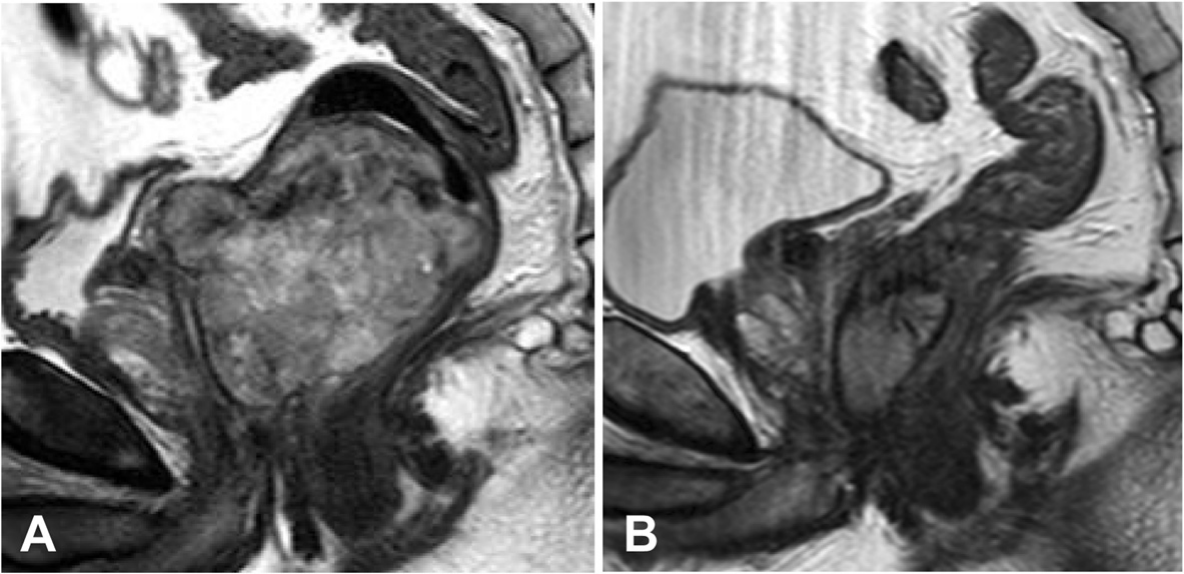
Sagittal view of pelvic MRI. **a** Before imatinib treatment, an 80-mm tumor was observed on the anterior wall of the lower rectum. **b** After 6 months treatment with imatinib, the tumor decreased to 37 mm

**Fig. 2:**
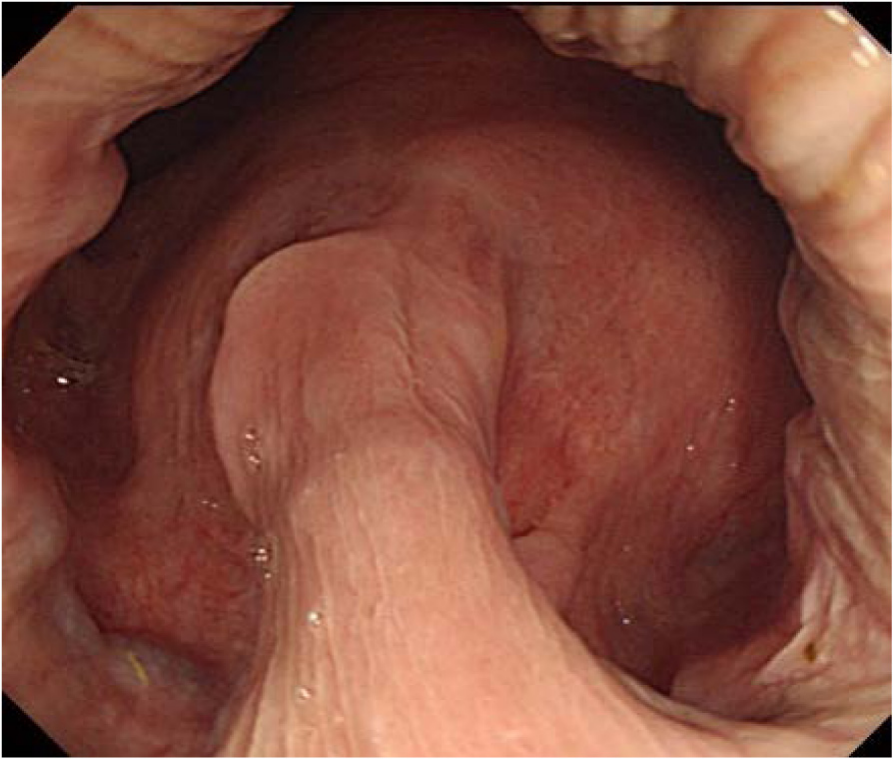
Colonoscopic image before imatinib treatment. A large submucosal tumor was observed on the anterior wall of the rectum just above the dentate line

**Fig. 3:**
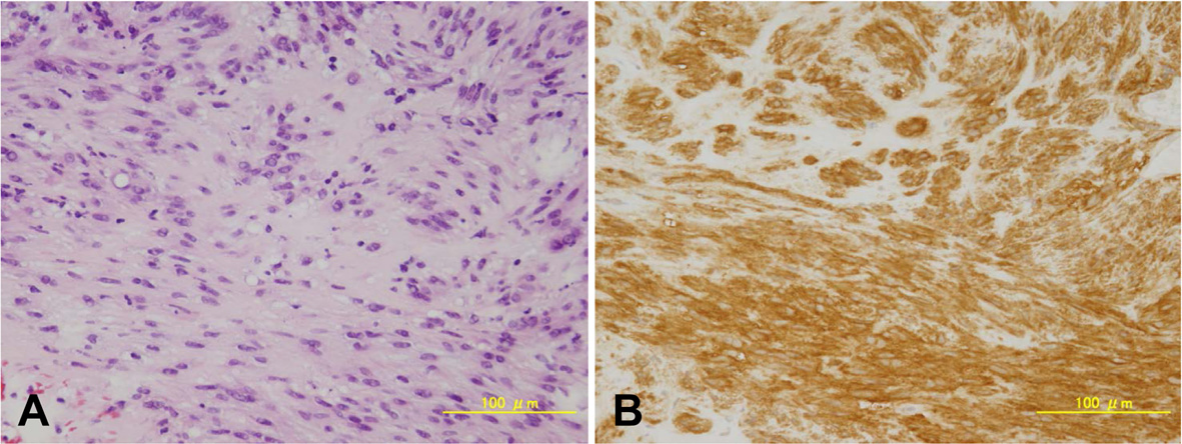
Core needle biopsy of the tumor. **a** Hematoxylin and eosin staining. **b** Immunohistochemical staining for c-kit antigen (CD117)

A 46-year-old man was referred to our hospital because of a 2-month history of anal pain and hematochezia. He also complained of constipation, malaise, and body weight loss of 10 kg in 1.5 years. Computed tomography (CT) and magnetic resonance imaging (MRI) demonstrated a tumor measuring 80 mm in maximum diameter on the anterior wall of the lower rectum and several enlarged regional nodes (Fig. 1a). No distant metastases were found. Colonoscopic examination revealed a large submucosal tumor on the anterior wall of the rectum just above the dentate line (Fig. 2). Core needle biopsy of the tumor revealed bundles of spindle cells with positive immunohistochemical staining for c-kit antigen (CD117) and CD34, but negative for other differentiation markers such as desmin and S-100 protein (Fig. 3a, b). The immunohistochemical findings led to the diagnosis of a rectal GIST. Neoadjuvant imatinib treatment (400 mg/day) was introduced, and soon after initiation, anal pain was relieved. MRI after 1 month of treatment showed marked shrinkage of the tumor to 52 mm, as well as the lymph nodes. After treatment for 3 and 6 months, the tumor decreased to 44 and 37 mm, respectively (Fig. 1b). CT examination revealed no distant metastases. As a result of the dramatic tumor shrinkage with neoadjuvant treatment and almost maximum tumor response, we decided to operate at this point. The adverse effect of imatinib was fatigue.

**Fig. 4:**
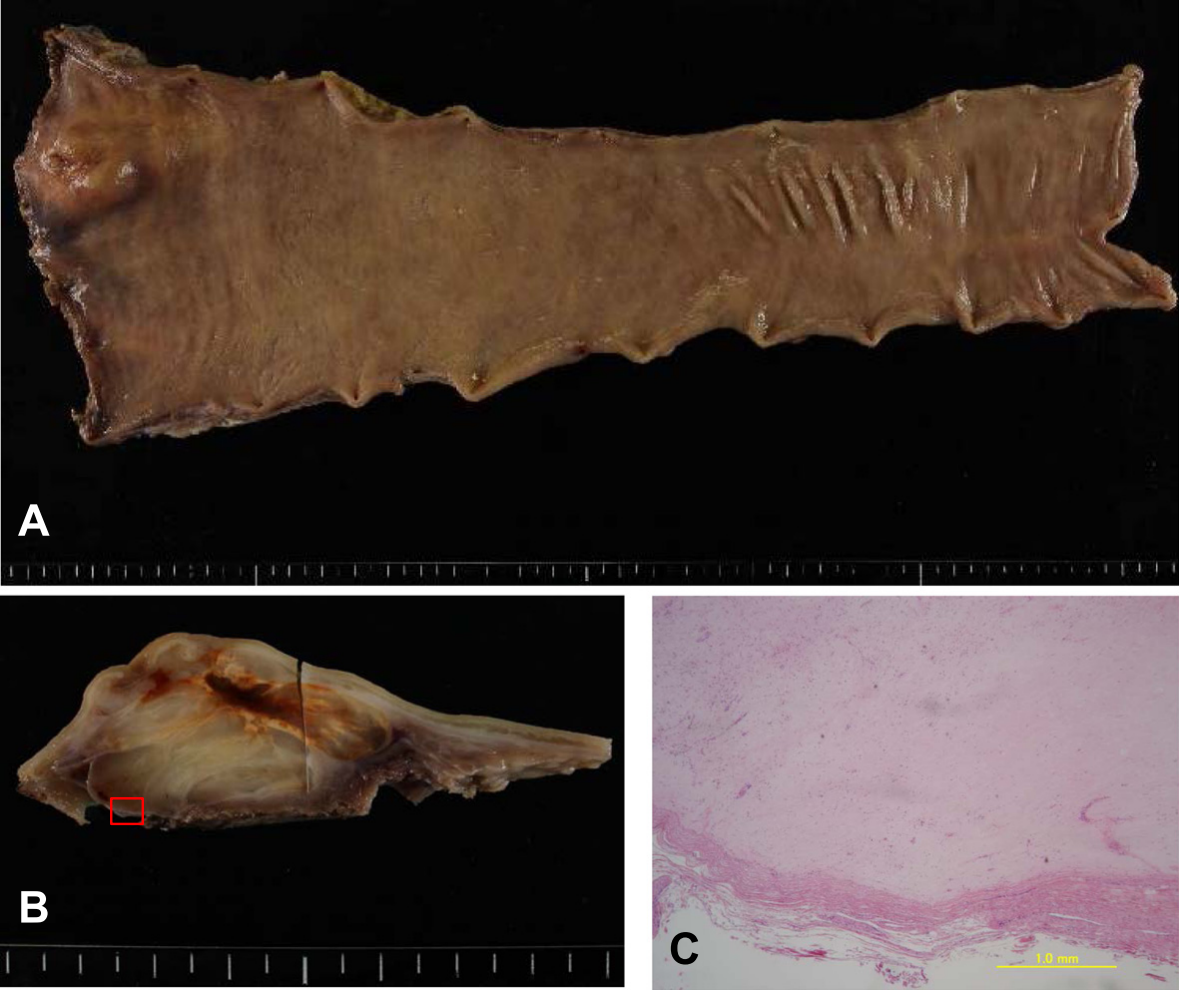
Pathological examinations. **a** Gross appearance of the resected specimen. The tumor measured 43 × 35 mm in size, and a 10-mm distal resection margin was secured. **b** Cross-sectional view of the tumor. Magnified view of the square area is shown in (**c**). **c** Histological examination (hematoxylin and eosin staining). All of the tumor cells caused hyaline degeneration

After 7 months treatment with imatinib, the patient underwent laparoscopic low anterior resection with total mesorectal excision, coloanal anastomosis, and diverting ileostomy. Imatinib treatment was stopped 3 days before surgery. Gross appearance of the specimen showed a submucosal tumor measuring 43 × 35 mm, with a 10-mm distal resection margin (Fig. 4a, b). Histological examinations revealed that the tumor spread within muscularis propria and protruded into submucosal layer in its anal side, with negative resection margins, and demonstrated an excellent response to imatinib with almost complete tumor necrosis (Fig. 4c). Metastasis was not found in the 11 retrieved lymph nodes. Imatinib treatment resumed on postoperative day 7, but was interrupted 1 year later because of fatigue. The ileostomy was reversed 3 months after the surgery, and the patient is currently alive without any evidence of recurrence 37 months after surgery.

### Discussion

We safely performed laparoscopic anus-preserving surgery after neoadjuvant imatinib treatment for a large rectal GIST. The primary treatment of choice for patients with localized GISTs is complete surgical excision with negative microscopic margins [10, 11]. However, in the restricted space of the pelvis, complete resection of a large rectal GIST is difficult and often necessitates abdominoperineal resection, with or without adjacent organ resection. To reduce the size of the tumor, thereby lowering the risk of rupture during surgery, and increasing the likelihood of anus preservation, we introduced neoadjuvant imatinib treatment in our patient. Many studies have shown the effectiveness of imatinib in the treatment of GISTs since the first report in 2001 [5]. Randomized clinical trials have reported response rates of ~70 % in patients with unresectable or metastatic GISTs [12, 13]. Another study demonstrated that, among 161 patients with locally advanced GISTs who received neoadjuvant imatinib treatment, 129 patients (80.1 %) had a partial response and only two (1.2 %) showed disease progression during treatment [14]. As for rectal GISTs, several reports demonstrated that neoadjuvant imatinib treatment improved R0 resection rates and decreased the risk of postoperative morbidity [6, 7]. There seems to be no worldwide consensus, however, as to the indications for neoadjuvant imatinib treatment, and it is not recommended in the clinical practice guidelines for GIST in Japan [15]. In contrast, in the National Comprehensive Cancer Network guidelines, it is recommended that preoperative imatinib treatment should be considered if abdominoperineal resection is necessary to achieve a negative resection margin, or if the surgeon feels that multivisceral resection may be required [10]. In the European Society for Medical Oncology guidelines, neoadjuvant imatinib is recommended as a standard treatment if R0 surgery can be achieved through function-sparing surgery in the case of cytoreduction, and if the surgeon believes that the procedure is safer after cytoreduction [11].

The clinical response to imatinib is reported to depend on GIST genotype. Mutations in KIT exon 11 are the most common type (65.8–73.1 %), followed by no mutations (15.2–16.4 %) and mutations in KIT exon 9 (8.2–8.4 %). Each of the other mutations comprises ~1 % of the total. Mutations in KIT exon 11 correlated with better response rates compared with no mutations and mutations in KIT exon 9, and the response rates were 69–71.7 %, 25–44.6 %, and 34–44.4 %, respectively [16, 17]. In patients with KIT exon 9 mutations, high-dose imatinib (800 mg/day) resulted in improved response rates compared with standard-dose therapy (400 mg/day) [17]. Hence, although mutational analysis is crucial to make a clinical decision about neoadjuvant therapy, the patient required as rapid a start of imatinib treatment as possible, without mutational analysis. This is partly because of the anal pain and the wish to preserve the anus, and partly because of unavailability of the high-dose imatinib regimen in Japan. Instead of the mutational analysis, we carried out close monitoring of the tumor response by MRI, particularly in the early phase of the treatment.

The appropriate timing of surgery is unknown. In randomized clinical studies, the cumulative incidence of response almost reached a plateau after treatment for 6–8 months, and disease progression occurred in some patients even in this period [12, 16]. Furthermore, it was demonstrated that the median time to best response was 3.5 months, and little tumor shrinkage was obtained after 9 months of treatment [18]. In the present case, although dramatic tumor shrinkage was obtained after 1 month of treatment, only a little tumor shrinkage was observed after 3 months. This represented almost maximum tumor response; therefore, we decided to operate at this point. Surgery should be done promptly when tumors become safely resectable, before disease progression.

Several approaches exist for resection of rectal GIST, but the principle of surgery is complete resection with an intact pseudo-capsule [10, 11]. Local resection, such as transanal, trans-sacral, or transvaginal, may be suitable for small tumors, but for larger ones, radical resection, such as low anterior resection is preferable. Several studies have demonstrated the association between local resection and local recurrence [7, 19]. One study showed that, among the 36 patients who underwent surgery for rectal GIST, all five who developed local recurrence had undergone local resection with positive margins, and without perioperative imatinib treatment [7]. Radical resection was more likely to have resulted in negative resection margins (R0) than local resection (13 of 15 vs. 11 of 21, *P* = 0.03), although the radical resection group had larger tumors. Another study also demonstrated that the local recurrence rate after local resection was 77 % compared with 31 % after radical resection, although the tumors were smaller in local resection group [19].

The remarkable response to imatinib enabled us to perform laparoscopic surgery safely. Several studies have demonstrated that laparoscopic resection for a small- or medium-sized gastric GIST is safe and effective when performed by an expert surgeon [20]. However, there are only a few reports of the effectiveness of laparoscopic surgery for a rectal GIST; partly because of the rarity of the disease [8, 9]. Although open radical resection for rectal GIST resulted in lower rates of involved resection margins and local recurrence than local resection, they still showed high values [7, 19]. This is at least partly because of the poor visualization in the deep narrow pelvis. Laparoscopy provides an excellent magnified view in the deep confined space of the pelvis; therefore, laparoscopic surgery should improve surgical outcomes and may be the preferred procedure in patients with small- or medium-sized rectal GISTs. Although we performed total mesorectal excision to dissect the enlarged nodes detected before the neoadjuvant treatment, the incidence of node involvement has been reported as 5.0–8.8 % [21, 22], and lymphadenectomy is usually not required [10, 11].

## Conclusions

Neoadjuvant imatinib should be a treatment of choice for large rectal GIST to increase the chance of anal preservation, and achieve surgical safety. When dramatic tumor shrinkage is obtained, laparoscopic surgery may be the preferred procedure.

## Consent

Written informed consent was obtained from the patient for publication of this Case report and accompanying images. A copy of the written consent is available for review by the Editor-in-Chief of this journal.

## Abbreviations

CT: computed tomography
GIST: gastrointestinal stromal tumor
MRI: magnetic resonance imaging
